# Synthesis of Cerium Oxide Nanoparticles Using Various Methods: Implications for Biomedical Applications

**DOI:** 10.3390/nano10020242

**Published:** 2020-01-29

**Authors:** Mpumelelo Nyoka, Yahya E. Choonara, Pradeep Kumar, Pierre P. D. Kondiah, Viness Pillay

**Affiliations:** Wits Advanced Drug Delivery Platform Research Unit, Department of Pharmacy and Pharmacology, School of Therapeutics Sciences, Faculty of Health Sciences, University of the Witwatersrand, 7 York Road, Parktown, Johannesburg 2193, South Africa; mpumelelo.nyoka1@students.wits.ac.za (M.N.); yahya.choonara@wits.ac.za (Y.E.C.); pradeep.kumar@wits.ac.za (P.K.); pierre.kondiah@wits.ac.za (P.P.D.K.)

**Keywords:** neurodegenerative disease, cerium oxide nanoparticles, Parkinson’s disease, oxidative stress, physicochemical properties, blood-brain barrier, synthesis methods

## Abstract

Cerium oxide nanoparticles have been used in a number of non-medical products over the years. The therapeutic application of these nanoparticles has mainly been due to their oxidative stress ameliorating abilities. Their enzyme-mimetic catalytic ability to change between the Ce^3+^ and Ce^4+^ species makes them ideal for a role as free-radical scavengers for systemic diseases as well as neurodegenerative diseases. In this review, we look at various methods of synthesis (including the use of stabilizing/capping agents and precursors), and how the synthesis method affects the physicochemical properties, their behavior in biological environments, their catalytic abilities as well as their reported toxicity.

## 1. Introduction

**Figure 1:**
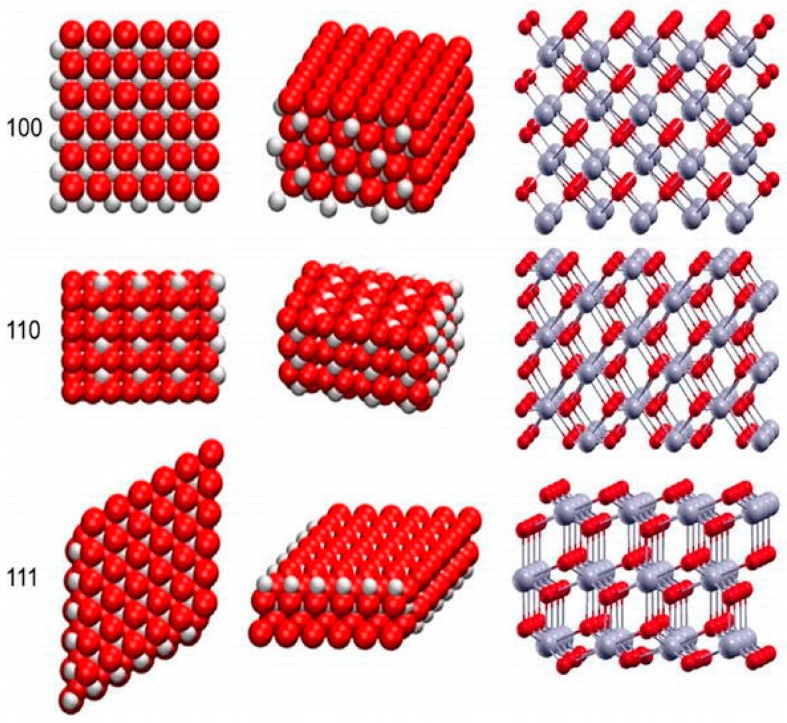
Crystal lattice and (100), (110), and (111) surfaces of cerium oxide nanoparticles [11], used with no changes under the terms of the Creative Commons Attribution 3.0 International License (Creative Commons Attribution 3.0 License).

During the latter years of the 1960s, scientists dedicated to miniaturized delivery systems introduced nanoparticle-based drug delivery systems and vaccines [1]. Since that application of nanoparticulates into medicine, the use of nanomaterials has yielded great advances in the diagnosis and treatment of numerous pathologies [2,3]. Cerium belongs to a class of lanthanide metals in the periodic table [4]. In oxide form, cerium has a fluorite structure. The nanoscale form, cerium oxide nanoparticles retain the fluorite structure with oxygen deficiencies. This yields cerium oxide nanoparticles with (CeO^2−x^) vacancies which provide sites for reduction-oxidation reactions. The arrangement of the surfaces of the fluorite structure determines the catalytic performance of the nanoparticles. The (100), (110), and (111) are the best possible surfaces on a cerium oxide nanocrystal (see Figure 1 below) [5]. These represent the lattice arrangement of ions (Ce^3+^ and O^2−^) in the fluorite structure. The (111) and the (100) possess the o-terminal endings, while the (110) arrangement exposes the Ce center and the O ions [5,6]. These properties enable these nanoparticles to be very useful in industrial applications such as the removal of carbon monoxide, hydrocarbons, and nitric oxide species from the exhaust gas. This property is facilitated by the ability of cerium to occur in trivalent (Ce^+3^) and tetravalent (Ce^+4^) states [7,8]. Cerium oxide nanoparticles have been shown to mitigate oxidative stress damage, which has been linked to the development of neurodegenerative diseases such as Parkinson’s and Alzheimer’s disease. The ability of cerium oxide nanoparticles to switch between valency states enables them to mimic specific enzyme functions such as superoxide dismutase (SOD), Catalase (CAT) and phosphatase, oxidase peroxidase, and phosphotriesterase [9,10].

**Figure 2:**
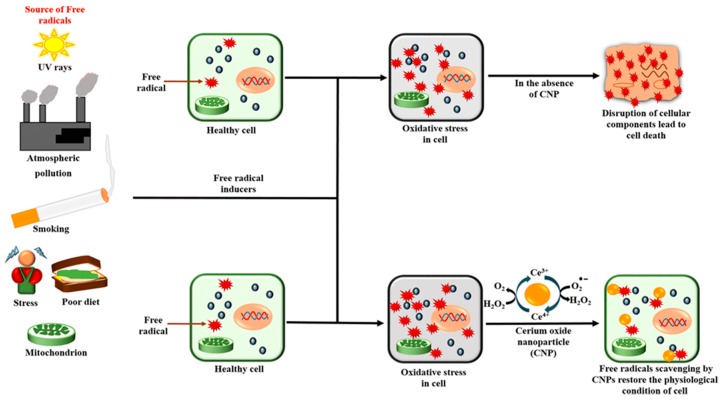
A depiction of some of the causes of oxidative stress and the redox action of cerium oxide nanoparticles (CNPs) [15], used with no changes under the terms of the Creative Commons Attribution 4.0 International License (http://creativecommons.org/publicdomain/zero/1.0/).

Cerium oxide nanoparticles are cost-effective and maintain their catalytic properties under harsh environments (Figure 2). Although they are not naturally occurring enzymes, they possess powerful SOD-like activities. Their ability to scavenge reactive oxygen species in an enzyme-like fashion renders them a suitable biocompatible alternative for natural bio-scavengers such as superoxide dismutase [12]. The surface properties of nanoparticles impact on their enzyme mimetic activities. The formation of nanostructures of cerium oxide alters the oxygen non-stoichiometry ratio of Ce^3+^/Ce^4+^ on the surface of nanostructures. The activity of cerium oxide nanoparticles correlates with the oxygen non-stoichiometry of nanoparticles. The SOD-like enzyme-mimetic activities of cerium oxide nanoparticles have been reported to be dependent on the Ce^3+^ fraction [10,13]. Another report evaluates the surface properties of cerium oxide nanoparticles using x-ray photoelectron spectroscopy (XPS) and UV-vis. The study illustrates that treatment of the nanoparticles with hydrogen peroxide decreases the Ce^3+^/Ce^4+^ ratio which correlates directly with the loss of SOD-mimetic activity [13,14].

Cerium oxide nanoparticles have also shown catalase-mimicking activities. A recent study exploring the mimetic activities of these nanoparticles employed the pulsed electron evaporation as the method of synthesis. The catalase-mimicking activities were measured by the addition of hydrogen peroxide to the cerium oxide nanoparticle (CNP) suspension and was measured at the 380 nm absorption. Vazirov and coworkers suggested that surface doping of metals onto the CNPs increases the Ce^3+^/Ce^4+^ ratio and the number of vacancies on the nanoparticle surface [16]. This consequently increases the points of interaction and reaction on the surfaces of the nanoparticles. The catalase-like activities are dependent on the Ce^4+^ fraction. A study assessed the catalase-like activities of CNPs using the Amplex Red assay to detect the physiological levels/concentration of peroxide. The test demonstrated catalase-like activity in the suspension with high levels of Ce^4+^ compared to a suspension with high Ce^3+^ [14,17].

In general, phosphorylation and dephosphorylation mechanisms are very valuable in the regulation of critical physiological conditions. The process involves the addition and removal of phosphate groups for energy maintenance. This, in turn, is the fundamental process in the synthesis of adenosine triphosphate (ATP) molecule. Adenosine diphosphate (ADP) is the hydrolyzed form of ATP. Upon hydrolysis, energy (H^+^) is released and the phosphate is used in multiple cellular applications such as cellular physiological and metabolism. Cerium oxide nanoparticles and HMT-CNPs (Hexamethylenetetramine-cerium oxide nanoparticles) were tested for phosphate-like activity. HMT-CNPs decrease the levels of ATPs, thus exhibiting substantial ATPase (phosphatase) activity. This causes a decrease in cell viability which leads to an increase ADP, resulting in toxicity [16].

Moreover, pro and antioxidant properties are dependent on the method of synthesis, the ratio of +3/+4 and their ability to switch between the two species, which most studies have found to be the increase of this ratio that provides (+3) antioxidant properties after internalization of the nanoceria [9,18].

## 2. Synthesis of Cerium Oxide Nanoparticles and Their Properties

Studies have demonstrated different methods for the synthesis of cerium oxide nanoparticles for different applications. The differences in the conditions of synthesis influence the end product. This then implies that the resulting nanostructures will possess different physical/morphological as well as chemical properties, thus affecting the behavior of each. It is very important to consider the intended application of the nanostructures in order to determine the method of synthesis. The final features of the nanostructures are very important, especially in the medical application, as their properties affect the interaction at the biological interface [19].

While numerous studies have documented the therapeutic and beneficial effects of cerium oxide nanoparticles, some studies have suggested that cerium oxide nanoparticles may exhibit toxic and harmful effects on cells [20,21]. However, there are studies that show that the resultant properties such as pro/antioxidant and toxicity of cerium oxide are mainly dependent on synthesis conditions such as pH, temperature, and method of synthesis which confer behavior-altering physicochemical properties such as size/agglomeration, morphology, surface chemistry, and zeta potential [22,23,24,25]. These properties then dictate the behavior and therapeutic effectiveness upon application.

### 2.1. Size of Nanoparticles

In general, the size of nanoparticles enables the delivery of therapeutics across biological membranes without compromising the integrity of the membrane. Changes in nanoparticle sizes directly affect their characteristics and behavior. These changes influence biological parameters such as the biological half-life, diffusivity, and immunogenicity [26]. Decreases in the sizes of the particles increases the surface area to volume ratio. The large surface to volume ratio influences the catalytic properties of cerium oxide nanoparticles. The large surface area to volume ratio of minute nanoparticles enables particles to entrap free radicals on the surface [27]. Studies suggest that as the size of the cerium oxide nanoparticles decreases it promotes the reducibility and high oxygen storage capacity [28]. The increase in the oxygen vacancies enhances the movement of oxygen through the crystal lattice, thus assisting in the ability of ceria nanoparticles to oxidize or reduce molecules. It has been reported that a decrease in the size of cerium oxide nanoparticles correlates with an expansion of the lattice [15]. This arrangement increases the oxygen vacancies on the surfaces of the nanoparticles [29].

A study by Morones and coworkers demonstrated that the smaller the size of the nanoparticles the more potent the bacterial properties of silver nanoparticles [30]. Eriksson and coworkers demonstrated that cerium oxide nanoparticles between 3–5 nm offer exceptional antioxidative properties when doped into gadolinium for MRI imaging [26]. Studies have also identified size as one of the causes of nanoparticle toxicity. For these reasons, the size of nanoparticles is important in engineering the desired effects of nanotherapeutics. Zhang et al. were able to control the sizes of cerium oxide nanoparticles by adjusting a couple of parameters. In order to obtain particle sizes of between 3–12 nm, they adjusted the reaction times. However, in order to obtain larger nanoparticles, they adjusted temperature was adjusted between 400–800 °C for 30 min [29]. Dhall and Self suggested that careful consideration of the interaction of cerium oxide nanoparticles with the biological milieu is needed when synthesizing nanoparticles for therapeutic intervention [31]. It has also been suggested that the size of the nanoparticles contributes in the vascular clearance of cerium oxide nanoparticles by the immune system. “Bare” or un-coated cerium oxide nanoparticles tend to aggregate, which increases the size. However, applying a stabilizer prevents aggregation and increases the retention time in the body. This increase in size due to aggregation causes rapid clearance by the reticuloendothelial system [32]. Xu and Qu suggested that the body does not possess mechanisms for clearance of cerium oxide as it is not naturally produced in the human body [33]. However, when cerium oxide nanoparticles are ingested, they are not quickly absorbed and they are excreted via feces [32]. Negatively charged cerium oxide nanoparticles under the size of 6 nm have been also found to be excreted via the renal system [34].

### 2.2. Aggregation and Agglomeration of the Particle System

The use and meaning of the words ‘aggregation’ and ‘agglomeration’ are discipline-specific. Here we define ‘aggregation’ as strongly bonded or fused collection of particles while ‘agglomeration’ involves loosely clumped nanoparticles (or aggregates) held by weaker van der Waals forces. The biological environment and nanoparticle physicochemical characteristics (e.g., surface properties, particle size) are believed to influence the agglomeration and aggregation of the nanoparticles [35,36]. The Brownian motion is a major factor in this physical behavior as it causes continuous collisions between the particles. As a result of this agglomeration occurs as the energy of attraction exceeds the energy of repulsion [37]. The forces involved in the collisions include Born repulsion, diffuse double layer potential, and van der Waals attraction. It is also known that aggregate size in the solution depends on properties such as initial particle size and concentration [38]. Also, the aggregate size may vary among different particle types, zinc oxide nanoparticles (NPs) dispersed in aqueous solutions aggregate in a wide range of sizes [39,40,41] whereas TiO2 NPs showed a uniform distribution and agglomeration [42].

### 2.3. Particle Morphology

**Figure 3:**
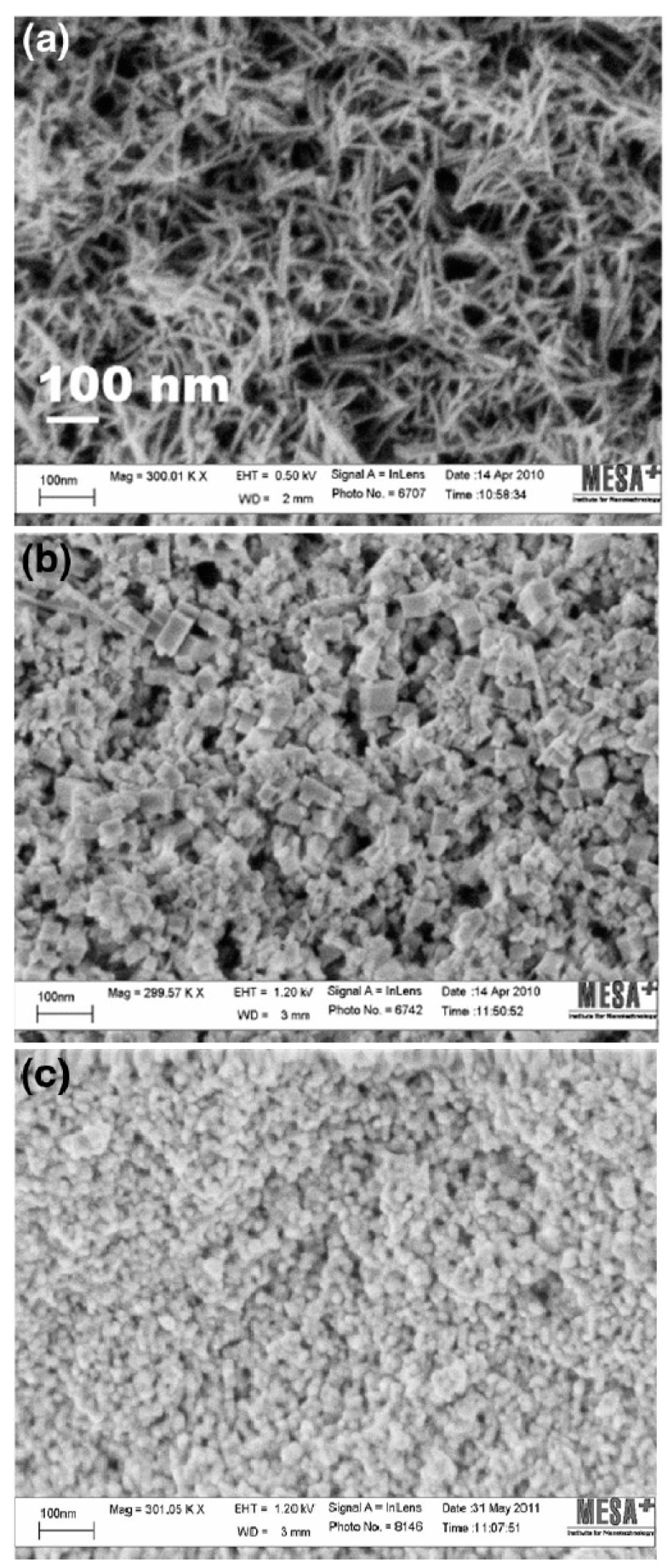
HRSEM images of cerium oxide nanoparticles in different morphologies; (**a**) nanorods, (**b**) nanocubes, and (**c**) nanospheres [19], used with no changes under the terms of the Creative Commons Attribution 4.0 International License (http://creativecommons.org/licenses/by/4.0/).

The particle morphology is one of the vital physical properties that are to be considered for efficacious nano-therapeutics in biological systems (Figure 3) [19]. Interaction between nanoparticles and biological components can be affected by the morphology of the nanoparticles. Studies have suggested that the method of synthesis may affect particle morphology. Nanoparticles exist in various shapes including spherical, polygonal, cube, or rod shapes. Nanoparticles with sharp edges are less biocompatible as they may inflict mechanical damage on cell membranes and organelles. Particle shape is known to play an important role in the fate and behavior of manufactured NPs into their environment. This could be as a result of the differences in diffusion rates of the material change with the aspect ratio of the material (e.g., higher drag on a tubular structure compared to a perfect sphere) or because of steric hindrance in the collisions as the morphology limits inter-particular interactions [43]. Several reports have addressed the role of shape and size on cellular internalization [44,45,46].

### 2.4. Chemical Composition

The chemical composition of the medium is known to have a crucial impact on the electrostatic surface charge of the nanoparticles, thereby affecting the rate at which these nanoparticles agglomerate/aggregate and thus affecting nanoparticle stability. Most of the manufactured nanoparticles nowadays are coated with surfactants to increase the stability of the suspension. The presence of a surface coating on manufactured NPs may significantly modify their surface chemistry, compared with the uncoated equivalents [18,47]. Similar kinds of changes may happen when pristine particles are treated with complex media such as humic acids. For example, adsorption of humic acids on the surface of small aggregates of silver NPs is known to result in the disaggregation of the NPs [48]. Similarly, using stabilizers on the surfaces of cerium oxide nanoparticle limits inter-particle interactions which may result in aggregation [21]. The authors also suggest that the application of stabilizers prevents agglomeration-induced toxicity. Andreescu and colleagues suggested that the environment to which cerium oxide nanoparticles are exposed to may affect their surface chemistry. The interaction of the hydrogen ions in the solution as well as the oxygen atoms in the facets of the lattice alters the surface chemistry of the nanoparticles and consequently alters their catalytic abilities (see Figure 1). The authors also suggest that the nanoparticle surface chemistry is highly sensitized to components of an environment such as pH, proteins, oxidizing, and/reducing agents [49]. Szymanski and company showed that in a biological environment, cerium oxide nanoparticles showed different oxidation states in different parts of the cells or organelles. Nanoparticles in the intracellular environment were shown to have a high Ce^3+^/Ce^4+^ ratio compared to those in the extracellular environment. This suggests that a noticeable reduction of the cerium oxide nanoparticles occurs in the cells. The authors postulate that differences in pH of the endocytic vesicles (pH = 7.4) versus lysosome (pH = 4.5) may also be the reason for these differences in surface chemistry [50]. Qi and company synthesized PPEG (Phosphonated-Polyethylene glycol oligomers) surface modified nano-powder for re-dispersion in aqueous solutions. The authors showed that the coated cerium oxide nanoparticles were successfully re-dispersed in various organic solvents such as acetone, chloroform, and ethanol. The complexing of the oligomer increased the stability of these nanoparticles when compared to their uncoated/bare counterparts [51]. These examples suggest that the chemical composition of the environment to which cerium oxide nanoparticles are exposed, impacts the surface chemistry and ultimately the behavior of the nanoparticles.

### 2.5. Surface Chemistry and Physical Properties

Redox state: Redox reactions (oxidation and reduction processes) occurring on the surface of nanoparticles result in an altered crystalline nature. For example, cerium oxide NPs. Cerium occurs in both trivalent (III) as well as tetravalent (IV) states and has the unique ability to switch readily between these two states [18,52]. This low energy change confers unique catalytic properties on the cerium oxide nanoparticles [53]. However, it is known that the oxidation state is spatially variable within an individual particle and is dependent on size [54], so understanding of the redox mechanism on the particle surface is crucial.

Zeta potential: ‘zeta potential‘ is the potential difference between the dispersion medium and the layer of fluid attached to the dispersed particle and is often used as an analog for colloidal stability, although this is only relevant where NPs are charge stabilized.

Solubility/Dissolution: Dissolution is a dynamic process in which the contents of the dissolving substance migrate from the surface to the bulk solution through a diffusion layer [55]. The thermodynamic parameter that controls this process is described as solubility [56]. Metal-based NPs such as zinc oxide are known to dissolve quickly and release ions that are themselves known to be toxic. Thus, the extent of dissolution and the relative toxicities of both the nanoparticulate and dissolved forms need to be considered to better understand the potential NP effects on organisms over time [57].

Surface functionalization of nanostructures has been used to synthesize non-toxic, stable, and biocompatible nano-therapeutics for applications in various conditions. Polymer chemistry in nano-therapeutics is a means of improving bioavailability, biocompatibility, solubility, as well as bio-retention of water-soluble and insoluble therapeutics for biomedical applications [58,59]. A number of these biodegradable polymers have been applied in the synthesis of multiple stable cerium oxide nanoparticle-systems [60,61]. Cerium oxide nanoparticles can be covered by hydroxyl groups. Therefore, biocompatible and biodegradable polymers that intrinsically possess hydroxyl moieties are capable of stabilizing the CNPs.

Applying the polymers as capping/stabilizing agents can assist in logically controlling the diameter of NPs [62]. The major challenge of suspending nanoparticles in solution is the ability to maintain their physicochemical properties. The surface charge of the nanoparticles, the low volume to surface area ratio of the nanoparticles causes them agglomerate, thus increasing the size of the particles from nano-range to micro-range [63,64]. This then negatively impacts on their therapeutic properties. In order to mitigate this, the surface coating is often employed to prevent the particles from agglomerating. This application of biocompatible coating enhances the solubility of the nanoparticles. Applying these coatings onto the surface of particles increases the stability and mitigates the toxicity of nanoparticles for efficient therapy.

### 2.6. Precipitation Method

Numerous studies have looked at various precursors to synthesize cerium oxide nanoparticles using the precipitation method, Table 1. Changes in any of the factors such as precursor, pH, and temperature alter the physicochemical properties of the cerium oxide nanoparticles. Researchers have investigated and reported on the precipitation method for the synthesis of cerium oxide nanoparticles using various chemical ingredients. Some of the reagents that are used in the precipitation method include precursors such as cerium nitrate hexahydrate and sodium hydroxide [26,65,66].

Metal oxide powders are acquired by the addition of a ligand such as ammonia into solution of metal ions. In order to obtain a precipitate from this solution or reaction, the point of solubility must be surpassed. In a study by Chen and Chang, the effect of the atmosphere, as well as the reaction temperature in the synthesis of cerium oxide nanoparticles, were studied. The precursor aqueous cerium nitrate and ammonia water were used. The temperature, stirring rate, the atmosphere in the reaction chamber, and the pH of 9.5 was set. The results suggest that increases in temperature change the morphology of the nanoparticles from cubic to hexagonal. Increases in oxygen content or/and decrease in temperature of the reaction atmosphere decreases the size of the nanoparticles [68].

The application of surfactants, capping, or surface doping agents has also been demonstrated in controlling the physicochemical and even catalytic properties of nanoparticles. In a study by Ramachandran and coworkers sought to synthesize cerium oxide nanoparticles with a preferred morphology. It is to be noted that controlling the resulting morphology of nanostructures can be a challenging task. In order to synthesize the nanoparticles, the pH was varied between one and 12 in order to investigate its effect on nanoparticle properties. The synthesis was carried out using cerium nitrate, polyvinyl/pyrrilidone as a surfactant. The synthesis was conducted at different pH ranges of between nine and 12. The XPS results suggest that at the pH of 12, the Ce^4+^ ion was abundant on the surfaces of the particles in the sample. The results also showed that the size of nanoparticles with spherical morphology decreases as the pH increases towards the pH 12 [69].

Cerium oxide nanoparticles possess catalytic, optical, and luminescence properties that have been assessed in imaging for therapeutics. A number of studies have proven that doping metals onto the surfaces of nanoparticles may enhance the properties of the nanoparticles. In order to improve these imaging qualities while maintaining the oxygen vacancies that provide them with their catalytic properties, Nanda sought to surface modify cerium oxide nanoparticles. In their study, they used ammonia-induced ethylene glycol-assisted precipitation to synthesize Samarium-doped cerium oxide nanoparticles (Sm-doped cerium oxide nanoparticles). The dynamic light scattering results from this study revealed that the nanoparticles had a hydrodynamic size of 236 nm for the Sm-doped nanoparticles. This was larger compared to that of the (6-{2-[2-(2-methoxy-ethoxy)-ethoxy]-ethoxy}-hexyl) triethoxysilane (MEEETES)-Sm-doped nanoparticles (MEEETES-Sm-doped nanoparticles), which has a hydrodynamic size of 116.4 nm [70]. Along with the doped cerium oxide nanoparticles, a hydrophilic MEEETES was used as a functional moiety on the surface. The application of biocompatible surface modifying moiety, MEEETES inhibits aggregation and enables dispersion in media. Their results comparing the zeta potential of the nanoparticles at day 0 and at day 180 showed that the zeta potential changed from between −11 and −12 mV at day 0 to between −14 and −13 mV on day 180 [71]. These insignificant changes over a period of 180 days suggests that the zeta potential of nanoparticle can be stabilized over time by the application of stabilizing/doping agents. These studies also suggest that using hydrophilic moieties such as MEEETES on the surface of the nanoparticles may influence not only the hydrodynamic size but also the stability of the zeta potential.

The pH is a vital factor to consider when synthesizing cerium oxide nanoparticles for therapeutic applications. The pH of environment to which the nanoparticles are applied can affect the catalytic properties of the nanoparticles. Therapeutic properties of dextran-coated nanoparticles were investigated in an in vitro study using osteosarcoma cells. The synthesis was carried out using cerium nitrate solution, 0.1 M dextran and ammonium hydroxide. The resulting product consisted of spherical particles of sizes between 3–5 nm. The investigation was carried out in an acidic pH of 3, 5, and 6 to replicate the acidic cancerous conditions, neutral pH of 7, and in an alkaline pH of 9. Characterization of the dextran-coated nanoparticles using XPS showed that the nanoparticles with the Ce^3+^ oxidation states on there were synthesized using this method. However, the effect of the pH was observed when the surfaces of the nanoparticles had Ce^4+^ in abundance at pH 6 and pH 7. The pH was also shown to have an effect on the zeta potential. The recorded zeta potential at pH 7 was 0.58 mV compared to that of 16.68 mV at the pH of 6 and 6.32 mV at pH 9. A study by Yan and company suggests that altering the synthesis pH can alter the morphology and increases the surface area of the nanoparticles. This shows that applying a capping agent such as dextran and controlling the synthesis conditions such as pH may yield cerium oxide nanoparticles with predetermined properties and may enhance the therapeutic properties [72,73,74].

The abovementioned studies of cerium oxide nanoparticle synthesis using the precipitation method show that a number of studies use cerium nitrate hexahydrate as a precursor for synthesis. The morphology was predominantly spherical. Employing the precipitation method produced nanoparticles with sizes 3–27 nm. The use of capping agents/solvents/doping agents narrowed the polydispersity of the nanoparticles. The studies also suggest that the synthesis conditions also play a vital role in the resulting nanoparticles. Therefore, careful control of the synthesis conditions such as temperature, concentration of the reactants, pH, atmosphere, as well as stabilizing agents influence the properties of nanoparticles when employing the simple and economic precipitation method.

### 2.7. Microemulsification Method

Micro-emulsion is a method that utilizes a polar aqueous medium (water) and a non-polar aqueous medium (oil) in the presence of a surfactant. This method produces nanoparticles of controlled size and structure. The application of this method has yielded a number of insoluble products such as nanoparticles, Table 2, quantum dots of metal oxides, metals, silica, as well as lanthanide fluorides [75,76,77,78].

Properties of nanoparticles are crucial in their function. Some of those properties are surface area and oxygen storage capacity. Nanoparticles with high surface area and high oxygen storage capacity enhance the catalytic properties of cerium oxide nanoparticles. However, increased temperature treatment of between 600–800 °C increases particle size thus decreases the surface area. Bumajdad and coworkers sought to synthesize cerium oxide nanoparticles with exposed surfaces and high thermal stability. The microemulsion method included sodium bis(2-ethylhexyl) sulfosuccinate (AOT), di-n-didodecyldimethylammonium bromide (DDAB), and DTAB+Brij 35 as surfactants. The results suggested that the application of surfactants in the synthesis process yielded cerium oxide nanoparticles with high surface area compared to the surfactant-free synthesis. Moreover, application of surfactant such as DDAB increased the surface area and produced cerium oxide nanoparticles which are stable at high temperatures. The use of cerium nitrate hexahydrate and cerium chloride heptahydrate as precursors in the synthesis of nanoparticles, solvents, and surfactants were used at the temperature of 50 °C. After calcination at 400 °C, the XPS results suggest that the surface of the nanoparticles consists of both Ce^4+^ and Ce^3+^. Upon calcination, the Ce^4+^/Ce^3+^ ratio increased [75].

In a study by Kockrick and coworkers, the team employed the microemulsion method to synthesize cerium oxide nanoparticles. The nanoparticles were calcined at a temperature of between 100–600 °C and the results suggest a size-dependent catalytic activity. The increase in temperature also aligns with an increase in size of the nanoparticles and a decrease in catalytic activity. The size was narrowly controlled using surfactants to between 6–16 nm [76]. In another study, the pH treatment of the nanoparticles was conducted and the zeta potential was measured. The zeta potential of the nanoparticles synthesized using this method was negative compared to a positive zeta potential of nanoparticles obtained from hydrothermal synthesis. The use of NH_4_OH resulted in the zeta potential of −16.26 mV. The study also found that positive zeta potential assists in protein adsorption as opposed to a negative zeta potential [78].

Arya and coworkers used cerium oxide nanoparticles in primary cortical cells as a means of protection against apoptosis via mitochondrial membrane depolarization. The authors also looked at the NADH/NAD+ and ATP concentration post cerium oxide nanoparticle treatment. Precursors such as cerium nitrate hexahydrate and hexamethyltetraamine were used in the microemulsion method of synthesis. The results reveal that spherical nanostructure with a diameter of between 7–10 nm was synthesized. The cortical primary cultures were treated with cerium oxide nanoparticles and the results suggest that cerium oxide nanoparticles prevented apoptosis by stabilizing the mitochondrial membrane potential [79].

Masui and coworkers prepared ultrafine cerium oxide nanoparticles with narrow size distribution and increased oxidative activity using the microemulsion method. The narrow size distribution was achieved by controlling the concentration of starting reagents. The ultra-fine nanoparticles synthesized by this microemulsion were not found to have the quantum-size effect. This was seen when the particle size had no direct and indirect optical energy gaps [80].

Cimini and coworkers investigated the neuroregenerative properties of anti-Aβ-conjugated polyethylene glycol (PEG)-coated cerium oxide nanoparticles in a model of oxidative stress-induced Alzheimer’s disease. The XPS results showed that the amine-functionalized nanoparticles had mixed oxidation state (Ce^4+^/Ce^3+^) on the surface. The zeta potential of amine-functionalized was +16 mV for bare cerium oxide nanoparticles and −37 mV for PEG-functionalized nanoparticles [81].

These studies suggest that the temperature, capping agents, and pH in the synthesis of cerium oxide nanoparticles using the microemulsion method of synthesis can control and narrow the particle size, morphology, and catalytic activity of cerium oxide nanoparticles. The various studies mentioned above show that the particle size was limited to between 2–21 nm. The predominant shape of the nanoparticles is seen to be spherical.

### 2.8. Hydrothermal Method

Particle sizes of the cube-shaped nanoparticles increases as the concentration of the solvent increased from 10–15 M. The hydrothermal method used produced rod-shaped nanoparticles at temperatures between 70–100 degrees Celsius as shown in Table 3. At a constant temperature of 100 degrees Celsius, the size of the nanosized particles decreased. Upon characterization of the nanoparticles using the temperature program reduction (TPR), the results showed an increase in the catalytic activity of nanorods versus nanocubes. This was due to the high concentration of surface oxygen on the surfaces of the nanoparticles. The high Ce^3+^ on the surfaces of the nanoparticles was confirmed by XPS analysis [82].

In a study that applied the hydrothermal method, Tok and coworkers sought to investigate the use of cerium hydroxide and/or cerium acetate as precursors. Thermal degradation and the duration of synthesis on the shape and crystallinity were investigated. The authors explored the effects of acidic (pH 4) and basic (pH 10) pH on the synthesis using either precursor prior to hydrothermal treatment. The results suggest that the crystalline morphology of the cerium oxide nanoparticles synthesized from both precursors yielded cubic structured nanostructures across different time-points (0 h, 6 h, 12 h, 18 h, and 24 h). At the 24 h time-point, the results suggest that the cerium acetate-derived/synthesized nanostructures were larger compared to the cerium hydroxide-synthesized nanostructures. Thermal treatments of cerium hydroxide-synthesized nanostructures at 500 °C and 1000 °C for 2 h grew the crystalline size to 8.8 and 47.4 nm, respectively from 5–6 nm. Thermal treatment of the cerium acetate-synthesized grew the crystalline size to 17.7 and 53.6 nm, respectively from 10–15 nm. The result also showed that in both precursor-derived nanostructures, agglomeration was observed [83].

Masui and coworkers investigated the application of citric acid as a protectant against particle growth. The surface of the particles was homogeneously covered with citric acid. Large particles formed prior to hydrothermal treatment when using Ce (III) salts were smaller in comparison with synthesis involving citric acid. This is because of the adsorption of citric acid onto the surface of the nanoparticles, thus controlling the nucleation process. The particle sizes of 5 nm were obtained from this method, with less agglomeration observed [80].

Cerium oxide nanoparticles with different morphologies were synthesized using hydrothermal synthesis. Different synthesis parameters such as temperature and concentrations were applied in order to manipulate the morphology of the resulting nanoparticles. The resulting nanostructures were nanocubes, nanorods, nano-octahedral, and submicronic cerium oxide nanoparticles. Thus, suggesting that altering synthesis parameters does alter the morphology and size of the nanoparticles. An analysis of the nanoparticles using XPS showed an oxidation state of Ce^3+^ on the surfaces of less than 1% [84].

In a different study, Zhang and coworkers synthesized degradable and stimuli-responsive polymer-coated cerium oxide nanorods for cancer therapy. The authors used dithio-polydopamine for the coating of the nanorods. The hydrothermal method of synthesis using cerium nitrate in mili-Q water (18.2 MΩ). In order to target cancerous cells, a lactose derivative moiety was attached to the surface of the nanoparticles. The resulting nanoparticles (spherical, cube, and rods) had hydrodynamic radii of 370, 194, and 192 nm and a set potential of −9.98, −10.86, and −5.9 mV [85].

Singh and coworkers evaluated the morphology and size-dependent cellular internalization at the nano-bio interface. The synthesis method used was hydrothermal synthesis. Varying concentrations of cerium nitrate hexahydrate (0.45–0.6 M) in deionized water were used. The results suggest that the resulting nanoparticles had a diameter of 3–5 nm. The results also suggest that the concentration altered the shape of the nanoparticles from spherical to nanorod to nanocube [86].

A myriad of morphologies can result from employing the hydrothermal method. Depending on the type of solvents, stabilizing agents, and synthesis conditions, resultant nanoparticles possess different properties.

### 2.9. Green Synthesis Routes

The green synthesis method is an environmentally innocuous technique that employs naturally occurring substances such as plant extracts, sugars, biodegradable polymers, and microorganisms in the synthesis of nanoparticles, Table 4. These naturally occurring components function as reducing as well as capping agents [87,88].

In a study by Thovhogi and coworkers, physicochemical properties of cerium oxide nanoparticles synthesis from Hibiscus Sabdariffa flower extract. The green synthesis method yielded nano-spheres with a diameter of 3.9 nm. An XPS investigation of the oxidation states on the surface of nanoparticles post-functionalization was undertaken. The results revealed a low 3+/4+ ratio. This shows the ability of environmentally friendly-green synthesis to alter the physical properties of nanoparticles [89].

Cerium oxide nanopowder with spherical particle sizes of 25 nm was synthesized using fresh egg white as a capping agent. The fresh egg white was used to control the particle distribution of the nanopowder. The eco-friendly capping and stabilizing agent is bio-degradable and possesses numerous amino acids. The ovalbumin and lysosome are the two major proteins that are available on the egg white. The interaction of the egg white with water and its ability to associate well with metal ions make it suitable for application as a shape-controlling stabilizing agent. The cell-viability of periodontal fibroblast cells treated with a range of CNP concentration of between 12.5–800 µg/mL was done. The results showed no toxicity of CNP concentrations below 800 µg/mL [90].

### 2.10. Solvothermal Method

Solvothermal synthesis employs the use of organic solvents in a chamber under high pressure and temperature to produce nanomaterials of varying sizes, Table 5. Teflon-lined autoclaves are used as they can withstand high temperature and pressure. Properties such as size were regulated by manipulating the temperature, reaction time, the concentration of the reagents, as well as the type of solvent. The 1,4-butanediol prohibited particle growth because of its viscous nature compared to water. This suggests that the viscous nature of a capping agent/solvent influences particle growth upon nucleation [91,92].

Kar and coworkers sought to synthesize ultra-small, water-soluble cerium oxide nanoparticles with the application of ethylenediamine as a surface capping/stabilizing agent at room temperature. The relatively monodispersed nanoparticles of 2.5 nm diameter showed mixed valence on the nanoparticle surface. The ultra-small particle exhibited a mixed oxidation state/valence on the surface of the nanoparticles [94]. The use of a solvent controls the physical properties of the cerium oxide nanoparticles such as morphology, particle size, and crystal growth. In this study, solvents/media such as water, ethanol, and ethylene glycol at different compositions were used. These were used to control the morphology, oxygen storage capacity, and electrical properties of cerium oxide nanoparticles. Water produced plate-shaped nanoparticles, spherical-shaped cerium oxide nanoparticles with a decreased size of the nanoparticles were produced with a 70:30 (water:ethanol) and the 70:30 (water:ethylene glycol) which resulted in porous nanoparticles [93].

### 2.11. Sol-Gel Method

Precursors such as cerium nitrate hexahydrate Ce(NO)_3_.6H2O undergo express hydrolysis, resulting in the production of a cerium hydroxide. The metal hydroxide then undergoes condensation thus forming a gel, Table 6. The gel is then exposed to a drying process to yield the final product [95].

Yu and coworkers used cerium (III) nitrate in a reaction with diphenyl ether as well as surfactants such as oleylamine to produce nanoparticles with spherical, wire, and tadpole morphology. The synthesis of nanostructures with different morphologies was achieved by using cerium nitrate and diphenyl ether in the presence of other surfactants. To obtain spherical morphologies oleylamine was used at the temperature of 320 °C. Nanowires were obtained from the application of the co-surfactants, oleylamine, and oleic acid at the temperature of 320 °C. Longer nanowires were synthesized by the addition of more oleic acid. Tadpole-like nanostructures were synthesized by increasing the amount of aleylamine in the co-surfactant mixture. The morphology and size of these nanostructures were synthesized/controlled by altering experimental parameters such as surfactant molar ratio as well as the duration of the reaction [95]. Another study investigated the effect of methanol as a solvent and the temperature of 400 °C on the size of nanoparticle morphology and optical properties. The results showed that the high ratio of UV to visible emission suggested good nanocrystal quality, i.e., good surface density. Nanoparticles with uniform spherical morphology with a size of 8 nm were synthesized [96].

A solution of cerium nitrate hexahydrate in a mixture of distilled water and Lu seeds and calcined at between 400–600 °C. The biosynthesis of these nanoparticles using the Lu seeds provided nanoparticles with altered physicochemical properties. Elahi and coworkers attached the 99Tc radio-labeller onto the nanoparticles in order to track the biological mobility/bioavailability of the nanoparticles in vivo. The results show on agglomerated morphology of the nanoparticles with an average size of 21–32 nm aligning with the calcination temperature of between 400–600 °C. These suggest that calcination, as well as the calcination temperature, has an effect on particle size. The smallest size showed no significant cytotoxicity on A549 cells. A bio-distribution to the kidneys and poor uptake by the stomach and thyroid were observed on an in vivo study using Wistar rats [97].

Another in vivo study on Wistar rats investigated the toxic effects of sol-gel prepared cerium oxide nanoparticles. The oxidative stress status, biochemical as well as hematological parameters were investigated. Pullulan was used as a capping agent to the cerium oxide nanoparticles. The resulting cerium oxide nanoparticles had a diameter of 12 nm. The results showed that pullulan-mediated cerium oxide nanoparticles possessed no cytotoxic effects upon acute administration on Neuro2A cells. There were also no toxic effects observed in the liver, spleen, and other organs. There was no damaged reported on the relevant antioxidant enzymes. The cerium oxide nanoparticles showed oxidative stress-protective properties and no hemotoxic effects or tissue damage upon histological analysis [98].

The green synthesis of cerium oxide nanoparticle-embedded hydrogel was conducted. The hydrogel was synthesized from carrageen as a stabilizing agent for the sol-gel synthesis of cerium oxide nanoparticles. Nourmohammadi and coworkers investigated the effectivity of carrageen hydrogel as capping agents on cerium oxide nanoparticles. They also investigated the cytotoxicity of these hydrogel-embedded nanoparticles on WEHI 164 cancer cells. The synthesis was conducted using cerium nitrate hexahydrate in double-distilled water and carrageen powder. The resulting nanoparticles possessed a spherical morphology with an average size of 34 nm. The results also showed no significant signs of toxicity in vitro even at high concentrations. However, the cellular metabolic activity was decreased with an increase in dose [99].

In a different study, starch was used as a capping agent on CNPs. The 6 nm spherical CNPs obtained were shown to have no cytotoxic effects in an in vitro study using the Neuro2A cell line [96]. The van der Waals forces between the particles increase the particle size during storage and upon contact with media. This causes the particles to be toxic to cells. Therefore, the application of starch as a stabilizing agent limited the inter-particular interactions which often result in aggregation and an increase in particle size. The cell-viability studies demonstrated no toxic effects on CNPs at a concentration of 5 µg/mL.

### 2.12. Ball Milling

A laboratory aluminar-zirconia ceramic mill and zirconia balls (ZrO2wt % 95%, diameter 0.5–0.3 mm, Mohs hardness 9) are used to create nanosized cerium oxide powder of 50 nm from cerium oxide powder with average particle sizes of 7.4 µm [100].

In a study by Yadav and coworkers, nanoparticles were produced from micron-sized cerium powder following 30 h of high energy ball milling, Table 7. The process yielded spherical nanoparticles with sizes between 8–12 nm with no defects. The conditions that yielded optimum results were noted to be 30 h of high energy ball milling at ice-cold temperatures [100]. Another study applied poly acrylic acid (PAA) to use as a dispersant of the cerium oxide nanoparticles. Normally, ball milling has been shown to produce nanoparticles with a diameter of 210 nm [101]. Li and coworkers used hydrated cerium carbonate and sodium hydroxide during high energy ball milling. In a different study, an organic base was used to synthesize cerium oxide nanoparticles with a nearly-spherical shape as opposed to an inorganic base during ball milling [102]. In this study, by He and coworkers, the use of PAA reduced the size of the nanoparticles from 128 nm to 50 nm and provided more stable cerium oxide nanoparticle slurry [103].

### 2.13. Flame Spray Pyrolysis

In an interesting study, Vassie and coworkers synthesized folic acid (FA)-coated cerium oxide nanoparticles by flame spray pyrolysis, Table 7. The aim was to investigate whether FA-coated cerium oxide nanoparticles can modulate intracellular activities and control ROS levels when compared to bare/uncoated cerium oxide nanoparticles. The liquid precursor, cerium 2-ethylhexanoate in xylene was used for the experiment. Using a syringe, the mixture was fed through the flame at a determined rate. Folic acid was also conjugated onto the nanoparticles to form FA-cerium oxide nanoparticles. The results show cytoplasmic localization of both the FA-cerium oxide nanoparticles and bare nanoparticles. There was also an increase in the uptake of the FA-coated nanoparticles in ovarian cancer cells that led to the induction of cell death via an increased concentration of ROS [105].

### 2.14. Reverse-Phase Evaporation

Liposome-entrapped cerium oxide nanoparticles were synthesized to enhance their therapeutic properties, Table 7. Cerium oxide nanoparticles with a size of 12 nm were loaded into liposomes. The sizes of nanoparticle-loaded liposomes were 230 nm. The liposomes enhanced the lipophilicity of the cerium oxide nanoparticles. The in vitro toxicity test was conducted on normal human dermal fibroblast (NHDF) cells. The study findings showed that the cerium oxide nanoparticle-loaded liposomes displayed colloidal stability and retained their antioxidant properties [106].

### 2.15. Reverse Micelle

An in vivo model of cardiotoxicity using rats was created to elucidate the ameliorative effects of cerium oxide nanoparticles, Table 7. The rat model was created by subcutaneous injection of isoproterenol hydrochloride (30 mg/kg) in saline. This study compared the ameliorative effects of Captopril and cerium oxide nanoparticles in oxidative-stress-induced cardiotoxicity. The cerium oxide loaded reverse micelles with sizes of 5 nm were synthesized. The results showed that cerium oxide-loaded micelles showed better prophylactic and ameliorative effects of cardio cytotoxicity compared with Captopril [107].

## 3. Toxicity and Cytotoxicity of Cerium Oxide Nanoparticles

The toxicity and cytotoxicity of cerium oxide has long been a point of contention among researchers exploring the application of cerium oxide nanoparticles in biomedicine. The lanthanide-derived nanoparticles have been applied immensely in industrial or non-medical applications. These applications include environmental chemistry, cosmetics, gas-emissions, fuel cells, oxygen sensors, and corrosion protectors [31,33].

Studies have suggested that the successful application of these metal oxides in the abovementioned fields may not infer positive application in biomedical systems. This is may be due to the complex nature of physiological systems. The properties of these nanoparticles and the manner in which interact with the biological milieu needs to be considered in order to synthesize biocompatible nanoparticles for biomedical application. This is because the properties and the manner in which these nanoparticles interact with the biological system may have toxic effects [108,109]. A study by Asati and coworkers investigated the surface charge-dependent internalization of polymer-coated cerium oxide nanoparticles into normal versus malignant cells. Transformed lung cells A549 and MCF-7 breast cells as well as untransformed HEK293 and H9c2 were chosen. Poly-(acrylic acid), aminated poly (acrylic acid), and/or dextran were applied as coating on the nanoparticles. These polymers furnished the nanoparticles with different charge characteristics such as negative, positive and neutral, respectively.

The internalization of the nanoparticles was observed in every cell type except the MCF-7 cell line. The negatively charged nanoparticles were internalized in the A549 cell line only and were not taken up by the MCF-7, thus the toxicity to the A549 lung carcinoma cells. Neutral nanoparticles were localized in the cytoplasm of all cell types in this experiment and were non-toxic. The study thus found that factors such as internalization, localization, and surface charge of the cerium oxide nanoparticles play an important role in the toxicity of cerium oxide nanoparticles [110]. In another study, alkaline-precipitation as well as the inverse micro-emulsion methods were employed to synthesize ultrafine cerium oxide nanoparticles. Li and coworkers synthesized water-insoluble (CeO2-P), water-soluble, dextran coated, PAA coated, and EDA coated nanoparticles were synthesized. The polymers provided different functional groups on nanoparticle surfaces such as –OH, –COOH, and -NH_2_. The results suggest a concentration-dependent cytotoxicity. The results also suggest that the “bare” cerium oxide nanoparticles (without functional groups) induced acute cytotoxicity on BGC-803 cell lines. The dextran, PAA, and EDA coated nanoparticles showed some viability as high concentrations (20 mg/mL) [14].

The toxicity of cerium oxide nanoparticles has also been attributed to their size. The smaller the size the more toxic they are. This has been suggested to be due to the large surface area to volume ratio. This means they possess more catalytic activity on the surface, thus making them very reactive and consequently toxic. Their size may also result in increased internalization into the cells which may result in toxicity. The tendency of the cerium oxide nanoparticles to agglomerate has also been suggested to be one of the causes of their toxicity [111]. The interaction of these metal oxide nanoparticles with their biological milieu may induce toxic effects. The synthesis method and the storage conditions have also been implicated in their toxicity [112]. These studies suggest that the physico-chemical properties of cerium oxide nanoparticles as well as the manner they interact with the biological environment may cause them to be toxic.

## 4. Conclusions and Future Perspectives

These studies clearly demonstrate the therapeutic effects and the behavior of cerium oxide nanoparticles in a bio-relevant environment. They also demonstrate the effects of synthesis and surface modification on the therapeutic effects of cerium oxide nanoparticles in the biological interface. This highlights the importance of the synthesis method or post-synthesis modifications of cerium oxide nanoparticles. The application of biocompatible and biodegradable capping and stabilizing agents clearly prove necessary in the synthesis of nanoparticles that are able to exert therapeutic effects. These capping agents have to be considered when synthesizing biologically-relevant nanoparticles in order to avoid unwanted interaction between the nanoparticles as well as between the nanoparticles and their environment. More studies looking into the therapeutic effects of cerium oxide nanoparticles in systemic conditions caused inter alia by oxidative stress, inflammation, and bacteria. Therapeutic effects of these nanoparticles in diseases that require tissue regeneration (scaffolds) need to be further explored.

## Figures and Tables

**Table 1:** Table showing the synthesis of cerium oxide nanoparticles using the precipitation method.

Capping Agent	Precursors	Particle Size (nm)	Morphology	Ref.
-	Cerium nitrate hexahydrate	9–18	Cubic Hexagonal	[67]
PVP	Cerium nitrate hexahydrate	27	Spherical	[67]
MEEETES Samarium	Cerium nitrate hexahydrate	10	Polyhedral	[68]
Ethylene glycol Samarium	Cerium nitrate hexahydrate	5–10	Square	[69]
Dextran	Cerium nitrate hexahydrate	3–5	Spherical	[70]
Sarium	Cerium nitrare hexahydrate	10–13	Spherical	[22,71,72]

**Table 2:** Table showing the synthesis of cerium oxide nanoparticles using the microemulsion method.

Capping Agent	Precursors	Particle Size (nm)	Morphology	Ref.
AOT DDAB DTAB Brij35	Cerium nitrate hexahydrate/Cerous chloride	6–13 (surfactant) 21 (No surfactant)	Cubic	[75]
OP-10	Cerium nitrate hexahydrate	2–6	-	[77]
Hexamethyl tetraamine	Cerium nitrate hexahydrate	7–10	Spherical	[79]
Ethylene glycol Aβ	Cerium nitrate hexahydrate	3–5	Spherical	[80]

**Table 3:** Table showing the synthesis of cerium oxide nanoparticles using the hydrothermal method.

Capping Agent	Precursors	Particle Size (nm)	Morphology	Ref.
-	Cerium nitrate hexahydrate	8–16	Cubes; rods	[79]
-	Cerium hydroxide/Cerium acetate	5–54	Cubes; amorphous	[81]
Citric acid	Cerium chloride	<5	Spherical	[82]
Trisodium phosphate dodecahydrate	Cerium nitrate hexahydrate	5–60	Rods; Cubes; octahedral	[83]
Dithio-polydopamine	Cerium nitrate hexahydrate	L = 60 d = 5.8	Rods	[80]
-	Cerium nitrare hexahydrate	3–5	Spherical; Cubes; Rods	[84]

**Table 4:** Table showing the synthesis of cerium oxide nanoparticles using the green synthesis method.

Capping Agent	Precursors	Particle Size (nm)	Morphology	Ref.
Acalypha indica	Cerium chloride heptahydrate	8–54	Eliptical spherical	[87]
Fructose; Glucose; Lactose	Ammonnium Cerium nitrate	2–6	Spherical/Agglomerate	[86]
Hibiscus Sabdariffa	Cerium nitrate hexahydrate	3.9	Amorphous	[89]
Egg White	Cerium nitrate hexahydrate	25	Spherical	[90]

**Table 5:** Table showing the synthesis of cerium oxide nanoparticles using the solvothermal method.

Capping Agent	Precursors	Particle Size (nm)	Morphology	Ref.
1,4-butanediol	Ceric ammonium nitrate	5–10	-	[92]
Ethylenediamine	Cerium nitrate hexahydrate	2.5–8	-	[91]
Ethylene glycol	Cerium nitrate hexahydrate	-	Plate; Spherical	[93]

**Table 6:** Table showing the synthesis of cerium oxide nanoparticles using the solvothermal method.

Capping Agent	Precursors	Particle Size (nm)	Morphology	Ref.
Diphenyl ether/oleylamine	Cerium nitrate hexahydrate	1.2–35	Spherical; tadpole; wire	[95]
Methanol	Cerium chloride heptahydrate	8–30	Spherical; Sheet-like	[96]
Lu seeds	Cerium nitrate hexahydrate	21–32	-	[97]
Pullulan	Cerium nitrate hexahydrate	-	-	[98]
Carrageenan	Cerium nitrate hexahydrate	18–60–	Spherical	[99]

**Table 7:** Table showing the synthesis of cerium oxide nanoparticles using the various synthesis methods.

Synthesis Method	Capping Agent	Precursors	Particle Size (nm)	Morphology	Ref.
Ball Milling	-	Cerium powder (5µm)	10	Spherical	[104]
Ball Milling	Tetrabutyl ammonium hydroxide	Cerium ammonium nitrate	5–56	Spherical	[100]
Flame spray pyrolysis	Folic acid	Cerium acetate hydrate	7	-	[102]
Reverse phase evaporation	Lipid anionic mixture	Cerium nitrate hexahydrate	12–230	Spherical	[103]
